# MiR-25 overexpression inhibits titanium particle-induced osteoclast differentiation via down-regulation of mitochondrial calcium uniporter in vitro

**DOI:** 10.1186/s13018-022-03030-7

**Published:** 2022-03-03

**Authors:** Weifan Hu, Yongbo Yu, Yang Sun, Feng Yuan, Fengchao Zhao

**Affiliations:** 1 Department of Orthopedics, The People’s Hospital of Jiawang District of Xuzhou, Xuzhou, 221000 People’s Republic of China; 2 Department of Orthopedics, The Affiliated Hospital of Xuzhou Medical University, 99 Huaihai Road, Quanshan District, Xuzhou City, Jiangsu Province 221000 People’s Republic of China

**Keywords:** Mitochondrial calcium uniporter, miR-25, Periprosthetic osteolysis, Osteoclast, Titanium particle

## Abstract

**Background:**

Mitochondrial calcium uniporter (MCU) is an important ion channel regulating calcium transport across the mitochondrial membrane. Calcium signaling, particularly via the Ca^2+^/NFATc1 pathway, has been identified as an important mediator of the osteoclast differentiation that leads to osteolysis around implants. The present study aimed to investigate whether down-regulation of MCU using microRNA-25 (miR-25) mimics could reduce osteoclast differentiation induced upon exposure to titanium (Ti) particles.

**Methods:**

Ti particles were prepared. Osteoclast differentiation of RAW264.7 cells was induced by adding Ti particles and determined by TRAP staining. Calcium oscillation was determined using a dual-wavelength technique. After exposure of the cells in each group to Ti particles or control medium for 5 days, relative MCU and NFATc1 mRNA expression levels were determined by RT-qPCR. MCU and NFATc1 protein expression was determined by western blotting. NFATc1 activation was determined by immunofluorescence staining. Comparisons among multiple groups were conducted using one-way analysis of variance followed by Tukey test, and differences were considered significant if *p* < 0.05.

**Results:**

MCU expression was reduced in response to miR-25 overexpression during the process of RAW 264.7 cell differentiation induced by Ti particles. Furthermore, osteoclast formation was inhibited, as evidenced by the low amplitude of calcium ion oscillation, reduced NFATc1 activation, and decreased mRNA and protein expression levels of nuclear factor-κB p65 and calmodulin kinases II/IV.

**Conclusions:**

Regulation of MCU expression can impact osteoclast differentiation, and the underlying mechanism likely involves the Ca^2+^/NFATc1 signal pathway. Therefore, MCU may be a promising target in the development of new strategies to prevent and treat periprosthetic osteolysis.

**Supplementary Information:**

The online version contains supplementary material available at 10.1186/s13018-022-03030-7.

## Background

Titanium (Ti) implants are commonly used in artificial joint replacements, and the influence of Ti on the periprosthetic environment and the subsequent effects on implant fate have been the subject of much research [1–3]. Sundfeldt et al*.* estimated that 10–15% of Ti prosthetics used in artificial joint replacement will loosen and require replacement within 15–20 years after implantation [4]. This is attributed to the inevitable wear of the materials caused by relative motion of the prosthetic and degradation/degeneration of the biomaterials, which will further generate wear debris particles. Particles from all implant types will induce the differentiation of osteoclasts and accelerate an inflammatory response, processes that eventually lead to periprosthetic osteolysis. The final results are joint loosening and prosthesis failures [5, 6].

Signaling mediated via calcium ions (Ca^2+^), an important messenger responsible for cell transduction, is involved in osteoclast differentiation. When osteoclast precursors are continuously stimulated by Ca^2+^ oscillation, the calcium-binding protein calmodulin undergoes a conformational transition, which further induces activation of its downstream effector proteins the Ca^2+^/calmodulin-dependent protein kinases (CaMKs) and calcineurin phosphatase, which is responsible for nuclear translocation of nuclear factor of activated T cells, cytoplasmic 1 (NFATc1), regulation of osteoclast- specific genes, and thus the promotion of osteoclast differentiation [7]. Moreover, Yang et al*.* demonstrated through down-regulation of regulator of G-protein signaling 10 (RGS10) that Ca^2+^ oscillations influence the process of osteoclast formation in vitro, resulting in increased bone mass, whereas osteoclast formation can be inhibited by removal of Ca^2+^ oscillation [8].

Mitochondria are the most important organelles that regulate Ca^2+^ signals in the cytoplasm [9]. Mitochondria can extract a large amount of Ca^2+^ from a specific subcellular structural domain and regulate Ca^2+^ oscillations, thereby controlling Ca^2+^ signal within cellular solute and affecting cellular functions [10]. Uptake of Ca^2+^ ions into mitochondria is mainly mediated by mitochondrial calcium uniporter (MCU), a calcium channel with high selectivity that excludes other cytosolic cations [11]. This behavior relies on membrane potential, through which Ca^2+^ rapidly moves into mitochondrial matrix along the electrochemical gradient, instead of energy supply from ATP hydrolysis as well as accompanied by co-transformation of other ions and molecules [12].

Mammalian MCU consists of MICU1/MICU2, MCUb, and EMRE (essential MCU regulator) subunits. The selective filter of MCU, which is formed by an α helix that combines Ca^2+^ with acidic amino acid rings, can distinguish Ca^2+^-binding sites, expose the negatively charged surface of the inter-membrane space, reduce the energy barrier for Ca^2+^ flux into the mitochondria, and achieve high selectivity for Ca^2+^ [11, 13, 14]. Through these actions, MCU controls Ca^2+^ flux across the mitochondrial membrane. When the cytosolic calcium concentration increases, MCU can rapidly sense, absorb and transport Ca^2+^ to mitochondrial matrix, directly influencing the frequency and amplitude of calcium signals within cytosol [15]. Therefore, the fate of cells can be mediated by suppressing or enhancing MCU levels to regulate cellular calcium signaling [16].

Because MCU is a ruthenium (Ru)-responsive channel, Ru compounds are commonly investigated as inhibitors of MCU that are able to efficiently enter cells [17]. However, Ru is a retardant for many cations, and thus, it may result in systemic dysfunctions related to these cations [18]. Other forms of MCU regulation have commonly involved posttranscriptional regulations. MicroRNAs (miRNAs) represent an important class of posttranscriptional regulators. Additional research revealed that when cardiac myocytes are exposed to H_2_O_2_, MCU expression is negatively correlated with miR-25 expression, and miR-25 overexpression can reduce mitochondrial Ca^2+^ overload and inactivate mitochondrial apoptosis, increasing the resistance of cardiac myocytes to oxidative damage [19]. In addition, a bioinformatics prediction algorithm has identified miR-25 as complementary to the 3′ untranslated region (UTR) sequence of MCU, with a 100% match of the informatics nucleotide at the 1075 site. Another experimental study showed that MCU expression is significantly negatively correlated with miR-25 expression, confirming that MCU is a direct target of miR-25 [20]. Thus, we chose miR-25 mimics as a potential regulator of MCU expression in the present study (Table 1).

**Table 1:** Experimental design

Group	Cells and treatments
A	RAW264.7 + miR-25 NC
B	RAW264.7 + miR-25 mimics
C	RAW264.7 + miR-25 NC + Ti particles
D	RAW264.7 + miR-25 mimics + Ti particles

Accordingly, we hypothesized that osteoclast differentiation could be mediated via regulation of calcium signaling achieved by inhibiting MCU. To test this hypothesis, we established an osteolysis model based on osteoclast differentiation of RAW 264.7 cells induced by Ti particles. miRNA-25 mimics were used to reduce MCU expression and thereby diminish mitochondrial Ca^2+^ oscillations. RAW 264.7 cells were transfected with miRNA-25 mimics, and the effects on MCU expression and Ti particle-induced osteolysis were investigated. The findings of this study are expected to provide a new strategy and target sites for the prevention and treatment of periprosthetic osteolysis.

## Methods

### Materials

Ti particles were obtained from ThermoFisher Scientific (purity 93%, diameter < 20 μm, USA). Phosphate-buffered saline (PBS) was purchased from Hyclone (USA). miR-25 mimics and miR-25 negative control (NC) (sequence: sense, AGUCUGGCUCUGUUCACGUUAC; antisense, GUAACGUGAACAGAGCVAGACU) were synthesized by GenePharm (Shanghai, China). Lipofectamine™ RNAiMAX (Invitrogen 2149333) was obtained from ThermoFisher Scientific. Fura-2 AM (S1052) was purchased from Beyotime Biotechnology (China). The tartrate-resistant acid phosphatase (TRAP) staining kit (387A) was obtained from Sigma-Aldrich (USA). The reverse transcription (RT) kit (MR101-01/02) and qPCR reaction kit (Q711-02) were obtained from Vazyme Biotech Co., Ltd (Nanjing, China). Paraformaldehyde and glycerin were purchased from Sinopharm Chemical Reagent Co., Ltd. (Shanghai, China). Triton X-100, Hoechst 33258, radioimmunoprecipitation assay (RIPA) lysis buffer and polymethylsulfonyl fluoride (PMSF) were purchased from Beyotime Biotechnology. The BCA protein assay kit (BL521A) was obtained from BIOSHARP (USA). The supplier information for primer sequences is presented in Additional file 1: Table S1, and that for antibodies is included in Additional file 2: Table S2.

### Preparation of Ti particles

Ti particles were first treated to achieve endotoxin removal as described previously [21, 22]. Briefly, Ti particles were baked at 180 °C for 6 h, then immersed in 75% ethanol solution, and shaken on a horizontal rotator for 48 h. After centrifugation, the collected Ti particles were re-dispersed in PBS for further sterilization treatment with high temperature and pressure. The Pierce LAL Chromogenic Endotoxin Quantitation Kit (ThermoFisher Scientific) was applied to ensure that endotoxin concentration among Ti particles was less than 0.1 EU/mL.

The final concentration of Ti particles used to induce osteoclast differentiation in this study was 0.1 mg/mL, in accordance with previous studies [21, 22].

### Cell culture and transfection

RAW 264.7 cells were obtained from the Cell Bank of the Chinese Academy of Sciences (Shanghai, China). Cells in the logarithmic phase of growth were considered most suitable for further experiments. When the cell density reached 40–50%, cells were transfected using Lipofectamine™ RNAiMAX (Invitrogen 2149333) according to the manufacturer’s instructions. Then the cells were cultured for 48 h after transfection before use in experiments. Osteoclast differentiation of RAW264.7 cells was induced by adding Ti particles (0.1 mg/mL) into the culture medium. The experimental groups are outlined in Table 1.

### Western blot analysis

After exposure of the cells in each group to Ti particles or control medium for 5 days, RIPA lysis buffer and PMSF were used to extract cellular protein. The total protein amount was assessed using a BCA protein assay kit, and 30 μg total protein was collected from each sample for further analysis. The collected proteins were separated on sodium dodecyl sulfate (SDS)-polyacrylamide gel electrophoresis (PAGE) gels and transferred to membranes (Millipore HATF00010). After blocking with nonfat dried milk for 1 h at room temperature, the membranes were incubated with primary antibody overnight at 4 °C. The second antibody was then added for 1 h of incubation at 37 °C. The specific bands were recorded using a chemiluminescence (ECL) detection system (Bio-Ras, ChemiDocXRS+, USA). The relative intensities of the protein bands were analyzed using ImageJ software (National Institutes of Health, USA), using β-actin as an internal reference.

### Determination of calcium oscillation

After exposure of the cells in each group to Ti particles or control medium for 24, 72, or 120 h, Fura-2 AM was added to the culture medium for a 45-min incubation at room temperature. The dual-wavelength technique was applied using a Luminescence Detector (Molecular Devices SpectraMax Rouge®i3, USA) with excitation wavelengths of 340 and 380 nm. The intensity ratio (340/380 nm) was recorded over time to reflect calcium oscillation.

### TRAP staining

After exposure to Ti particles or control medium for 5 days, RAW 264.7 cells were fixed and stained according to the instructions of the TRAP staining kit. The samples were observed under an optical microscope (Leica DMI3000B, Germany). Cells that were positively stained and had more than three nuclei were regarded as osteoclasts. Ten areas in each well were photographed for counting of osteoclasts within each field. The counts were repeated three times for each field and averaged.

### Immunofluorescence staining

Cells from each treatment group were fixed for 30 min in 4% paraformaldehyde and treated with Triton X-100 (0.1%). For staining, the cells were incubated with NFATc1 antibody at 4 °C overnight, then rinsed with PBS several times, and incubated with second antibody for 1 h in darkness at 37 °C. More specifically, the cells were fixed before cell membrane permeability (0.2% tritonX-100/PBS) was enhanced for being more conducive to the binding of NFATc1 antibody into the nucleus. Fluorescent staining is to verify the activation of NFATc1 in the nucleus.

The detailed information for the secondary antibodies used is provided in Additional file 2: Table S2. After staining with Hoechst33258 for 15 min in the darkness at room temperature, the staining slices were sealed using glycerin. The stained cells were observed using a fluorescence microscope (Olympus, Japan).

### Real-time PCR

After exposure of the cells in each group to Ti particles or control medium for 5 days, the total RNA from each sample was extracted using the Trizol method. Reverse transcription was carried out to obtain cDNA using the RT kit, and then the mRNA expression levels of MCU, NFATc1, CaMKII/IV and nuclear factor (NF)-κB p65 were determined using the qPCR reaction kit. The results were analyzed using a real-time fluorescent quantitative PCR instrument (LightCycler® 480II, Roche, Switzerland). Differences in the expression levels of target genes among the various groups were analyzed using the 2^−ΔΔCT^ method. The amplification conditions were set as: 94 °C for 10 min followed by 40 cycles of 94 °C for 20 s, 55 °C for 20 s, and 72 °C for 20 s. The primers used for reverse transcription were designed and synthesized by Sangon Biotech (Shanghai, China), and the sequences are listed in Additional file 1: Table S1.

### Statistical analysis

All data are presented as mean ± standard derivation (SD). Statistical analysis was carried out using GraphPad Prism 8 software (GraphPad, USA). Comparisons among multiple groups were conducted using one-way analysis of variance (ANOVA), and then Tukey test was used to identify significant differences between two groups. Unless otherwise noted, differences were considered significant if *p* < 0.05.

## Results

### Effect of miR-25 on MCU expression, calcium oscillation, and osteoclast formation

**Fig. 1:**
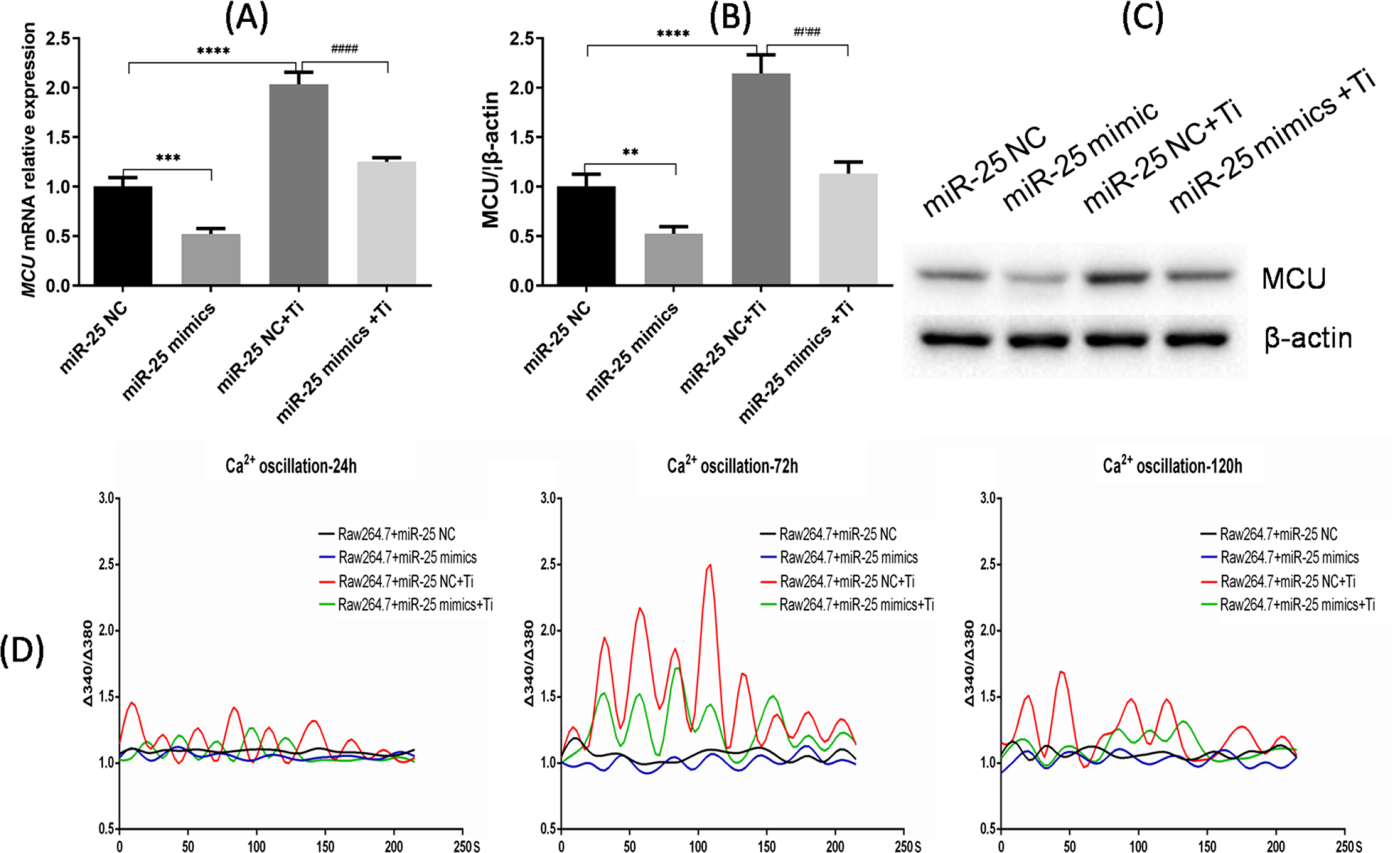
Reduced MCU expression influences Ca^2+^ oscillations in RAW 264.7 cells. **a** Relative MCU mRNA expression determined by RT-qPCR in RAW 264.7 cells of the different treatment groups after 5 days. **b** Western blot analysis of MCU protein expression and **c** quantified MCU expression relative to β-actin expression. **d** Ca^2+^ oscillation in RAW 264.7 cells of the different treatment groups at 24, 72, and 120 h. **p < 0.01, *** and ^###^*p* < 0.001, **** and ^####^*p* < 0.0001

To verify that miR-25 expression would lead to down-regulation of MCU expression during the process of osteoclast differentiation, we measured MCU expression in each treatment group. After 5 days in culture with control medium (no Ti particles), cells transfected with miR-25 mimics (group B) showed lower MCU mRNA and protein levels than those transfected with miR-25 NC (group A; *p* < 0.001; Fig. 1a–c). After 5 days in culture with medium containing Ti particles, the MCU mRNA level in cells transfected with miR-25 mimics and exposed to Ti particles (group D) was significantly lower than that in cells transfected with miR-25 NC and then exposed to Ti particles (group C; *p* < 0.0001; Fig. 1a). The same trend was observed for MCU protein expression (Fig. 1b, c). These results confirm that overexpression of miR-25 led to reduced MCU expression independent of the induction of osteoclast differentiation.

To examine the effect of MCU down-regulation on Ca^2+^ oscillations in the cells, we applied Fura-2 AM staining on days 1, 3, and 5 in the different treatment groups. As shown in Fig. 1d, Ca^2+^ oscillation was extremely low in the absence of Ti particles, whereas Ca^2+^ oscillation appeared with exposure to Ti particles. Importantly, the amplitude Ca^2+^ oscillation in cells of group C (miR-25 NC + Ti particles) was much greater than that in cells of group D (miR-25 mimics + Ti particles), indicating that down-regulation of MCU led to diminished Ca^2+^ oscillation during osteoclast differentiation.

**Fig. 2:**
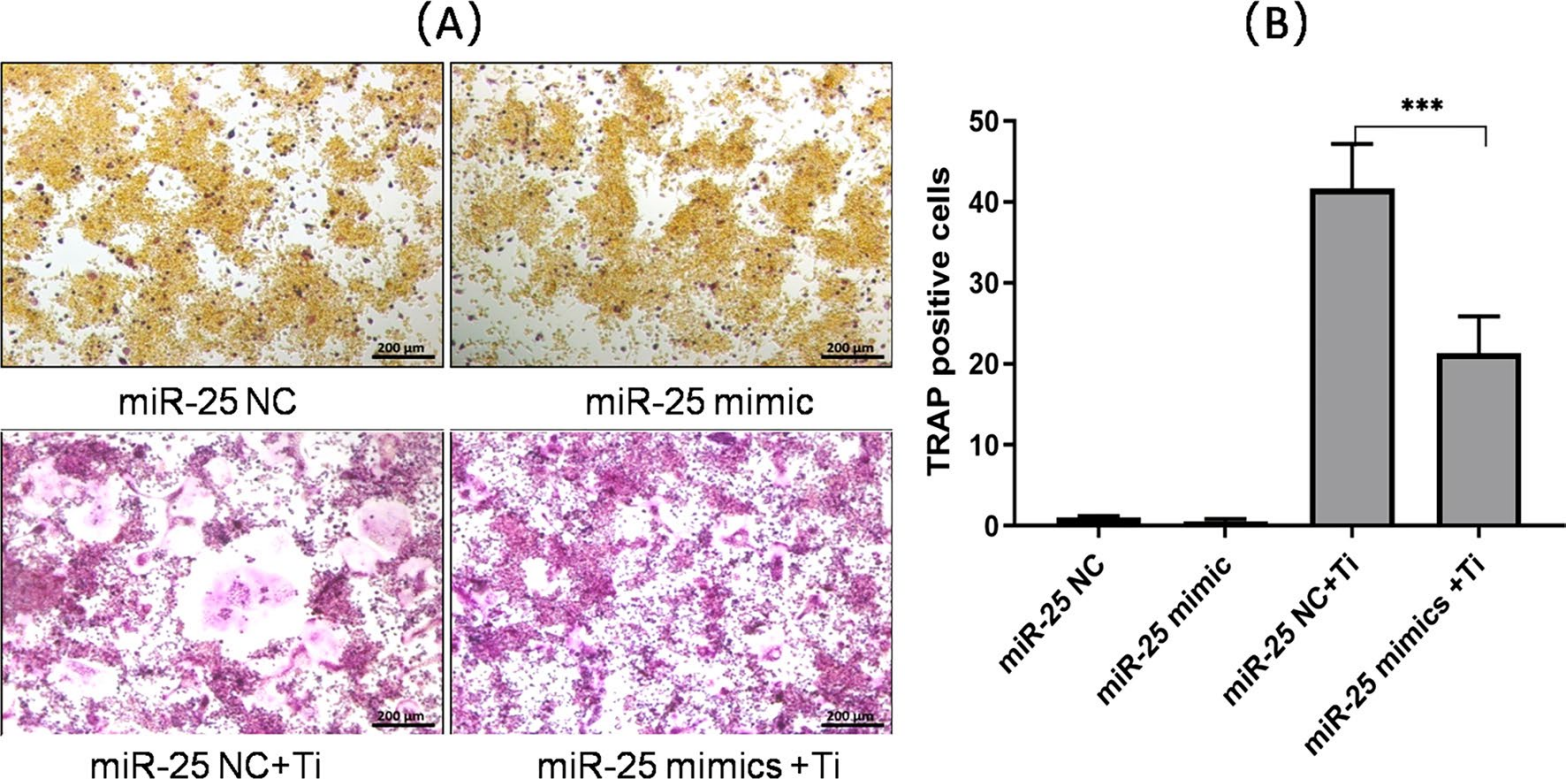
Down-regulation of MCU reduces osteoclast differentiation. **a** TRAP staining after exposure of the cells in each group to Ti particles or control medium for 5 days. Scale bars, 200 μm. **b** Numbers of osteoclasts counted in microscopic fields. ***p < 0.001

The effect of MCU down-regulation on Ti particle-induced osteoclast differentiation was investigated by TRAP staining. Upon exposure to Ti particles, RAW264.7 cells transfected with either miR-25 mimics or NC appeared as multinucleated osteoclasts with positive TRAP staining. However, with fewer and smaller osteoclasts were observed among the cells treated with miR-25 mimics and Ti particles (group D) compared with the cells treated with miR-25 NC and Ti particles (group C; Fig. 2a). The mean numbers of osteoclasts counted in the microscopic fields for groups C and D were 41.7 ± 5.5 and 21.3 ± 4.5 cells, respectively, indicating a significant 41.2% reduction in osteoclast formation with miR-25 mimics treatment (Fig. 2b).

### Effect of decreased MCU expression on Ca^2+^/NFATc1 signaling

**Fig. 3:**
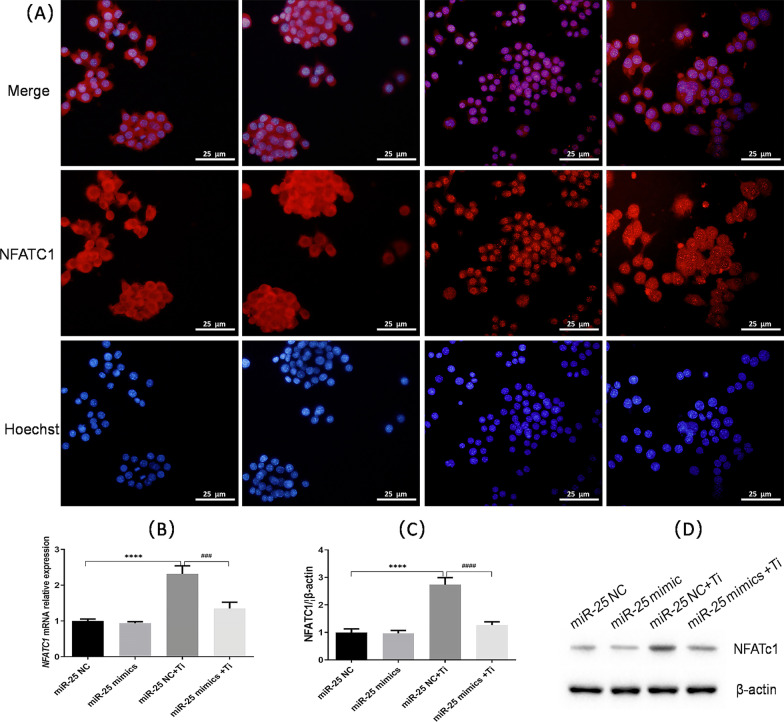
NFATc1 activation is diminished by reduced MCU expression. **a** NFATc1 activation determined by immunofluorescence staining of RAW 264.7 cells in the different treatment groups. **b** Relative NDATc1 mRNA expression determined by RT-qPCR in the different treatment groups. **c** NDATc1 protein expression determined by western blotting, and **d** quantitated NDATc1 protein expression, using β-actin as the internal reference

NFATc1 is an important transcription factor in the process of osteoclast differentiation that is activated by Ca^2+^ oscillation. To examine whether NFATc1 expression changed consistently with the reduced Ca^2+^ signaling observed after MCU down-regulation by miR-25 mimics treatment, the cells of the different treatment groups were subjected to immunofluorescence staining to detect NFATc1 activation. As shown in Fig. 3a, compared to cells treated with miR-25 NC and Ti particles (group C), those transfected with miR-25 mimics and then exposed to Ti particles (group D) exhibited reduced NFATc1 activation. Counting of NFATc1-positive cells in different microscopic fields yielded totals of 335.0 ± 53.0 positive cells in group C and 96.0 ± 38.6 positive cells in group D, indicating a 71.3% reduction in NFATc1 activation with MCU down-regulation (*p* < 0.01). Additionally, real-time PCR analysis showed decreased NFATc1 mRNA expression with MCU down-regulation induced by miR-25 expression (Fig. 3b). The results for NFATc1 protein expression also showed reduced NFATc1 expression in group D compared with group C (*p* < 0.0001; Fig. 3c, d).

**Fig. 4:**
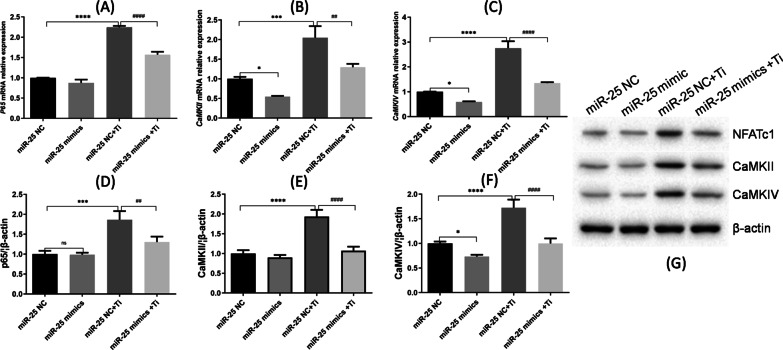
Effects of miR-25-mediated MCU down-regulation on the gene and protein expression levels of CaMKs. The relative mRNA expression levels of **a** NF-κB p65, **b** CaMK II and **c** CaMK IV, as determined by RT-PCR. Relative protein expression levels of **d** NF-κB p65, **e** CaMK II and **f** CaMK IV, as determined by western blotting and using β-actin as an internal reference

Variation in the intracellular calcium concentration supports the transport of transcription factors from the cytoplasm to the nucleus. During the process of osteoclast differentiation, in addition to NFATc1, NF-κB is another important transcription factor and indicator of inflammatory status. p65 is one of the key subunits of NF-κB. Therefore, we examined NF-κB expression in cells of the different treatment groups. Compared to cells in group C (miR-25 NC + Ti), those in group D (miR-25 mimics + Ti) showed significantly reduced levels of p65 mRNA expression (*p* < 0.0001, Fig. 4a) and NF-κB p65 protein expression (*p* < 0.01, Fig. 4d).

Considering our data showing that decreased MCU expression influences Ca^2+^ oscillation and subsequently osteoclast differentiation, we examined whether calmodulin activation of CaMKs was also affected. Indeed, cells in group D (miR-25 mimics + Ti) showed lower mRNA levels of CaMK II and CaMK IV than cells in group C (miR-25 NC + Ti; Fig. 4c). Moreover, similar results were observed for CaMK II and CaMK IV protein expression (Fig. 4e–g), indicating that miR-25 overexpression down-regulated the protein expression levels of these CaMKs.

## Discussion

Artificial joint replacement is an effective treatment strategy for many degenerative joint diseases and arthritis. It is highly successful during the early period post-operation; however, prosthesis loosening during later stages is a major concern. Such loosening is closely related to the increased need for artificial joint replacement. Several risk factors for revision joint surgery have been identified, including advanced age, osteoporosis, high cost, and physicians’ experience [23]. Thus, the prevention and treatment of aseptic loosening of prostheses is of great value for prolonging the service life of joint prostheses and reducing revision arthroplasty. Although the exact mechanism of aseptic loosening of prostheses remains to be determined, it is widely accepted that wear debris particles can induce osteoclast differentiation and osteolysis around a prosthesis [24]. As they are the only cells responsible for bone absorption in vivo, the differentiation and regulation of osteoclasts in the periprosthetic environment is of great significance for treating and preventing PPO.

Bisphosphonates have been used clinically to treat osteolysis induced by osteoporosis and some tumors, as they function to inhibit bone adsorption by osteoclasts. However, these drugs have troublesome side effects, such as gastrointestinal adverse reactions and nephrotoxicity, and accurate data supporting their application to prevent and treat osteolysis are lacking [25, 26]. Two alternatives, osteoprotegerin-Fc (OPG-Fc) and anti-RANKL (receptor activator of NF-κB ligand) therapy represent a major clinical strategy for inhibiting osteoclast differentiation. However, a wide variety of signaling molecules downstream of the RANKL signaling pathway are involved in complicated regulatory pathways with multiple functions [27]. Although clinical trials have not found an increased risk of bone malignancy or deterioration of metastatic bone tumors, anti-RANKL therapy in mice could cause dysfunction and even deficiency of lymphopoiesis, an effect that may be exacerbated by experimental and spontaneous tumor metastasis and more sensitive to chemical carcinogen [28]. Given the limitations of these therapeutic strategies, the Ca^2+^/NFATc1 signaling pathway has become a new target for inhibiting osteoclast differentiation. NFATc1 is known to play a key role in osteoclast differentiation, and with ectopic expression of the NFATc1 gene, bone marrow-derived mesenchymal cells (BMSCs)could differentiate into osteoclasts even without RANKL induction [29]. Moreover, Liu et al*.* established an in vitro osteolysis model induced by wear debris particles and found that regulation of the Ca^2+^/NFATc1 signaling pathway impacted osteoclast generation; specifically, Ti particle-induced osteoclast differentiation could be suppressed upon inactivation of NFATc1 using 11R-VIVIT peptide [21].

In the process of osteoclast differentiation, when pre-cells are stimulated, calcium channels on plasmatic membrane open, allowing the influx of extracellular calcium into cells, resulting in an increased intercellular calcium concentration. To maintain a calcium equilibrium, adjacent channels or organelles such as the endoplasmic reticulum further release calcium to the cytosol, resulting in nonlinear Ca^2+^ oscillations [30], which directly regulate the process of osteoclast differentiation. MCU plays a great role in this process. Our RT-PCR and western blot analyses confirmed that miR-25 overexpression led to reduced MCU gene and protein expression in RAW264.7 cells, both with and without Ti particle induction of their differentiation into osteoclasts, indicating that miR-25 is a regulator with strong specificity for MCU expression. Meanwhile, with the reduction in MCU levels, Ca^2+^ oscillations decreased and osteoclast formation was reduced, suggesting that down-regulation of MCU led to altered calcium signaling and, consequently, diminished Ti particle-induced osteoclast differentiation.

In the process of Ti particle-induced osteoclast formation in cells overexpressing miR-25, the mitochondrial Ca^2+^ influx was decreased and Ca^2+^ oscillations reduced. Accordingly, activation of NFATc1 was decreased in the absence of continuous stimulation of Ca^2+^ oscillation, and ultimately, osteoclast activity was diminished. In relation to this process, NF-κB and NFATc1 have a similar Rel homology domain (RHD) [31], and Ca^2+^ levels in the cytoplasm are suppressed and cannot be effectively activated to achieve successful nuclear transposition, resulting in reduced mRNA and protein expression of NF-κB p65 and decreased expression of osteoclast gene products. At the same time, due to their structural similarity, activated NF-κB and NFATc1 can directly interact to form a complex, which allows them to play a synergetic role in promoting transcription [32]. In the present study, Ca^2+^ oscillations were inhibited upon down-regulation of MCU, preventing this synergetic effect and finally reducing osteoclast differentiation. Of course, the NF-κB signaling pathway is also an important route for osteoclast differentiation, in which NF-κB is irreplaceable.

In terms of the Ca^2+^/NFATc1 pathway, CaMK family members (especially CaMK II and CaMK IV) are crucial molecules for the transfer of calcium signals that participate in the differentiation and functions of osteoclasts. Ca^2+^ oscillation induces a conformation change in calcium-binding proteins, leading to CaMK activation and regulation of osteoclast differentiation via the CaMK-CREB route. CaMKs can also regulate osteoclast-specific gene expression in cooperation with NFATc1. On the other hand, CaMKs and c-Fos synergistically act on the promoters of NFATc1 genes and trigger a self-amplification mechanism of NFATc1 [33]. In the present study, with the overexpression of miR-25, CaMK II and CaMK IV levels were reduced, resulting in decreased expression of osteoclast-specific genes, minimizing activation of the self-amplification mechanism of NFATc1 gene expression, and ultimately reducing osteoclast activity. In support of these findings, a recent study on neuronal injury caused by acute ischemia/reperfusion found that by blocking the activation of CaMK II/IKK/NF-κB signaling, down-regulation of NF-κB p65 subunits in mice could increase I/R-induced neuronal death, and up-regulation of CaMK II could increase activation of the NF-κB up-regulating pathway [34]. In consideration of the expression of CaMK and NF-κB observed in our study, we speculate that interaction of CaMKs and NF-κB may be another regulatory pathway for osteoclast differentiation.

In this study, transfection with miR-25 mimics decreased MCU expression in RAW 264.7 cells during the process of osteoclast differentiation induced by Ti particles, while also weakening Ca^2+^ oscillation and reducing NFATc1 activation and NF-κB expression. Furthermore, miR-25 expression also decreased CaMK (downstream signaling molecules) levels, inhibited osteoclast activity, and restricted osteoclast formation. These data provide experimental evidence for the theory that osteoclast differentiation can be inhibited through the Ca^2+^/NFATc1 signaling pathway. During this process, overload of intracellular calcium appeared due to the lack of MCU-mediated regulation of intracellular Ca^2+^, resulting in functional disorder and apoptosis of osteoclasts and causing osteoclast absorptive dysfunction. Further studies of the underlying mechanisms of these effects are warranted in support of the development of therapeutic strategies targeting MCU.

## Conclusion

In summary, we explored the effect of MCU expression on osteoclast formation. The data obtained in this study confirm that MCU is involved in the process of PPO induction by Ti particles. Transcription mediated by miR-25 can effectively act on MCU, inhibiting osteoclast differentiation possibly via the Ca^2+^/NFATc1 signaling pathway. However, this route for inhibiting osteoclast differentiation might also influence other cellular processes mediated by Ca^2+^ signaling, and thus, further studies are needed to determine the safety of this approach. Our analysis of miR25/MCU/Ca^2+^/NFATc1 interaction and its roles in osteoclast differentiation may provide a foundation for the future development of therapies to prevent and/or treat wear particle-induced PPO.

## Supplementary Information

**Additional file 1: Table S1.** Sequences of primers used in RT-PCR.

**Additional file 2: Table S2.** Details of antibodies used in this study.

## Data Availability

The datasets used and/or analysed during the current study are available from the corresponding author on reasonable request.

## Abbreviations

MCU: Mitochondrial calcium uniporter
NFATc1: Nuclear factor of activated T cells, cytoplasmic 1
CaMKs: Ca^2^^+^/calmodulin-dependent protein kinases
RANKL: Receptor activator of NF-κB ligand
OPG: Osteoprotegerin
PPO: Periprosthetic osteolysis
