# Supplementary material for: MiR-25 overexpression inhibits titanium particle-induced osteoclast differentiation via down-regulation of mitochondrial calcium uniporter in vitro
Source: J Orthop Surg Res. 2022 Mar 3;17:133. doi: 10.1186/s13018-022-03030-7 (PMC8895597; doi:10.1186/s13018-022-03030-7)
Supplement: Supplementary file 1 — Additional file 1: Table S1. Sequences of primers used in RT-PCR. [file 13018_2022_3030_MOESM1_ESM.docx]

**Table S1. Sequences of primers used in RT-PCR**

| **Name** | **Sequence** |
| --- | --- |
| Mcu-mouse-RT-F | AGCAGCATCAGCTTAACAAA |
| Mcu-mouse-RT-R | GTAGGTGACGGGCTCCAT |
| NFATc1-mouse-RT-F | ACCACTCCACCCACTTCTG |
| NFATc1-mouse-RT-R | GCTGCCTTCCGTCTCATAG |
| CAMKIV-mouse-RT-F | CTGTGCCTACGGACCTGA |
| CAMKIV-mouse-RT-R | AAAGACACTTCATCCCACC |
| CAMKII-mouse-RT-F | GTTGCTAACCCTCTACTTTCTC |
| CAMKII-mouse-RT-R | CTTTGGTGTCTTCGTCCTC |
| β-actin-mouse-RT-F | GTCCCTCACCCTCCCAAAAG |
| β-actin-mouse-RT-R | GCTGCCTCAACACCTCAACCC |
