# Supplementary material for: MiR-25 overexpression inhibits titanium particle-induced osteoclast differentiation via down-regulation of mitochondrial calcium uniporter in vitro
Source: J Orthop Surg Res. 2022 Mar 3;17:133. doi: 10.1186/s13018-022-03030-7 (PMC8895597; doi:10.1186/s13018-022-03030-7)
Supplement: Supplementary file 2 — Additional file 2: Table S2. Details of antibodies used in this study. [file 13018_2022_3030_MOESM2_ESM.docx]

**Table S2. Details of antibodies used in this study**

| **Name** | **Manufacturer information** | **Experiments** | **Mw (kDa)** | **Diluting Concentration for primary antibody** | **Host** | **Dilution ratio for second antibody** | **Manufacturer information of second antibody** |
| --- | --- | --- | --- | --- | --- | --- | --- |
| β-actin | abcam ab8227 | WB | 42 | 1:2000 | Rabbit | 1:4000 | biosharpBL003A |
| CaMKII | abcam ab22609 | WB | 50 | 1:500 | Mouse | 1:4000 | biosharpBL001A |
| CaMKIV | abcam ab3557 | WB | 55 | 1:2000 | Rabbit | 1:4000 | biosharpBL003A |
| MCU | abcam ab219827 | WB | 40 | 1:500 | Mouse | 1:4000 | biosharpBL001A |
| NFATcl | abcam ab25916 | WB | 100 | 1:500 | Rabbit | 1:4000 | biosharpBL003A |
| NFATcl | abcam ab25916 | IF | 100 | 1:20 | Rabbit | 1:200 | ServicebioGB21403 |
